# Ultrathin Mesoporous Metal‐Organic Framework Nanosheets

**DOI:** 10.1002/adma.202508105

**Published:** 2025-06-16

**Authors:** Yingji Zhao, Zhi Gao, Norman C.‐R. Chen, Yusuke Asakura, Ho Ngoc Nam, Quan Manh Phung, Yunqing Kang, Mandy Hei Man Leung, Dong Jiang, Lei Fu, Lijin Huang, Toru Asahi, Yusuke Yamauchi

**Affiliations:** ^1^ Department of Materials Process Engineering Graduate School of Engineering Nagoya University Nagoya 464–8603 Japan; ^2^ Faculty of Science and Engineering Waseda University 3‐4‐1 Okubo Shinjuku Tokyo 169–8555 Japan; ^3^ Jiangxi Province Key Laboratory of Functional Organic Polymers East China University of Technology Nanchang Jiangxi 330013 P. R. China; ^4^ Department of Chemistry Graduate School of Science Nagoya University Furu‐cho, Chikusa‐ku Nagoya 464–8602 Japan; ^5^ Institute of Transformative Bio‐Molecules (WPI‐ITbM) Nagoya University Furu‐cho, Chikusa‐ku Nagoya 464–8601 Japan; ^6^ State Key Laboratory of Geomicrobiology and Environmental Changes Faculty of Materials Science and Chemistry China University of Geosciences No. 388, Lumo Road, Hongshan District Wuhan 430074 P. R. China; ^7^ Australian Institute for Bioengineering and Nanotechnology (AIBN) The University of Queensland Brisbane QLD 4072 Australia; ^8^ Department of Convergent Biotechnology & Advanced Materials Science Kyung Hee University 1732 Deogyeong‐daero, Giheung‐gu, Yongin‐si Gyeonggi‐do Seoul 17104 South Korea

**Keywords:** 2D nanosheets, double soft‐template method, mesoporous structures, metal‐organic frameworks

## Abstract

Designing 2D mesoporous metal‐organic framework (MOF) nanosheets to overcome the limitations of bulk MOF counterparts, with a focus on enabling smooth mass transport, presents an attractive yet challenging endeavor. Here, a novel bottom‐up interface‐directed co‐assembly method is presented for the synthesis of ultrathin 2D mesoporous UiO‐66(Ce) nanosheets. The method utilizes an interface‐directed co‐assembly of amphiphilic perfluorooctanoic acid‐induced lipid bilayers and spherical micelles from PS‐*b*‐PEO block copolymers to form unique 2D sandwich‐like assemblies that guide the creation of 2D mesoporous UiO‐66(Ce). The resultant 2D mesoporous UiO‐66(Ce), with ≈23 nm pore diameters and a thickness that can be tuned from 3 to 150 nm, represents a substantial advancement in the application of MOFs for environmental remediation. As a model reaction, the U(VI) photoreduction benefits from the through‐mesopores of its 2D morphology, which are absent in previously reported UiO‐66(Ce), as they shorten the diffusion path, thereby improving mass transport and accessibility to active sites. This report demonstrates the significant role of existing mesopores in MOFs and the shape control of MOFs.

## Graphical abstract

A bottom‐up interface‐directed co‐assembly strategy enables the synthesis of ultrathin 2D mesoporous UiO‐66(Ce) nanosheets with tunable thickness and uniform mesopores. The enhanced mass transport and accessible active sites significantly improve performance, highlighting the synergistic effects of 2D morphology and mesopores in metal‐organic frameworks for applications in environmental remediation.

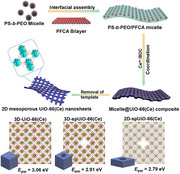

## 1 Introduction

Mesoporous materials have attracted significant interest owing to their notable characteristics, such as expansive surface areas, abundant pore volumes, modifiable nanostructures, and diverse compositions.^[^ ^1^, ^2^, ^3^, ^4^, ^5^ ^]^ The deliberate design and manipulation of the pore characteristics and shapes of mesoporous materials are crucial for specific applications.^[^ ^6^, ^7^, ^8^ ^]^ Among these, mesoporous metal‐organic frameworks (MOFs) have garnered significant attention owing to their numerous unique features and potential applications in various fields, including adsorption, separation, (electro)catalysis, and sensing.^[^ ^9^, ^10^, ^11^, ^12^, ^13^, ^14^ ^]^ The presence of large‐sized mesopores facilitates increased molecular accessibility, whereas small micropores in the MOF frameworks provide enhanced surface areas.^[^ ^15^ ^]^ Various mesoporous MOFs have been reported through bottom‐up self‐assembly methods.^[^ ^16^, ^17^ ^]^ Nonetheless, it is widely observed that most of these mesoporous MOFs predominantly appear in spherical and bulk solid forms.^[^ ^18^ ^]^

Considerable efforts have been made in the development of cutting‐edge 2D materials motivated by their attractive features, such as improved mechanical strength and superior electrical and optical performance, which are attributed to their low dimensionality and atomic‐scale thickness.^[^ ^19^, ^20^, ^21^, ^22^ ^]^ The 2D mesoporous MOFs combine the unique features of 2D morphology, mesoporous structures, and the inherent nature of MOFs. Incorporating mesopores also helps prevent the stacking of 2D nanosheets, creating pathways throughout their thickness that improve mass transport and increase the exposure of active sites.^[^ ^23^, ^24^ ^]^ Moreover, the lateral expansion of mesoporous MOFs leads to the creation of unique 2D ultrathin nanosheets, thus overcoming the limitations inherent to their bulk counterparts.^[^ ^25^ ^]^ However, due to limitations in synthetic protocols, current studies have predominantly yielded slit‐shaped porous 2D structures formed by aggregating numerous irregular mesoporous MOF nanoparticles with thicknesses around 30 nm, which lack complete interconnectivity.^[^ ^26^ ^]^ To the best of our knowledge, there are no successful reports on the preparation of continuous 2D mesoporous MOFs with thin thickness.

Rare earth elements have recently emerged as attractive metal centers in the construction of MOFs, owing to their distinctive electronic, optical, and catalytic properties.^[^ ^27^ ^]^ Cerium, as the most abundant rare earth element, provides unique advantages such as variable oxidation states (Ce^3+^/Ce^4+^), excellent oxygen storage and release capabilities, and redox activity, making Ce‐based MOFs exceptionally promising for applications in catalysis, environmental remediation, sensing, and energy storage.^[^ ^28^, ^29^ ^]^ Additionally, the integration of cerium into MOFs facilitates novel functionalities, including enhanced adsorption capacities, improved photocatalytic performance, and superior stability in various chemical environments, thus opening new avenues for the practical deployment of MOFs in technologically relevant fields.

**Figure 1:**
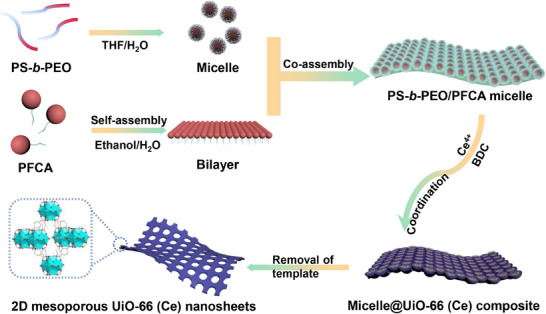
Schematic of the synthesis of the 2D mesoporous UiO‐66(Ce) nanosheets (2D‐mUiO‐66(Ce)).

This study aims to create ultrathin 2D mesoporous UiO‐66(Ce) nanosheets. We present a bottom‐up assembly method using spherical micelles derived from polystyrene‐*block*‐poly(ethylene oxide) (PS‐*b*‐PEO) block copolymers on the bilayer structure surface of amphiphilic perfluorooctanoic acid (PFCA)^[^ ^30^ ^]^ to extend the mesoporous architecture along the lateral direction (**Figure** **1**). Spherical micelles are assembled and arranged in a single‐layer pattern on the surface of 2D bilayers, ultimately providing hierarchical, sandwich‐like structures. The Tyndall effect was employed, using a low‐power laser, to verify the micellization of the block polymer with PFCA (Figure S1, Supporting Information). Upon introducing tetravalent cerium (Ce(IV)) species, weak coordination bonds are expected to form with the polyether oxide (PEO) segments, resulting in crown‐ether‐like complexes and the subsequent formation of cerium oxide clusters around the spherical micelles. Under the cooperative influence of salting‐in ions (ClO_4_^−^),^[^ ^31^ ^]^ the 2D sandwich‐like structures further guide the coordination reaction between cerium oxide clusters adsorbed within the PEO domains and terephthalic acid. After removing the soft templates, 2D mesoporous UiO‐66(Ce) nanosheets (abbreviated as 2D‐mUiO‐66 (Ce)) with a pore diameter of ≈23 nm and controllable thicknesses ranging from 3 to 150 nm are successfully synthesized. The developed 2D‐mUiO‐66(Ce) demonstrates enhanced U(VI) photoreduction efficiency compared to solid UiO‐66(Ce) (with predominant micropores) and mesoporous UiO‐66(Ce) (with hierarchical micro‐mesopores), both of which are in particle form, due to improved mass transport and reduced electron‐hole recombination. This highlights the significant impact of both morphology and mesopore effects.

## 2 Results and Discussion

**Figure 2:**
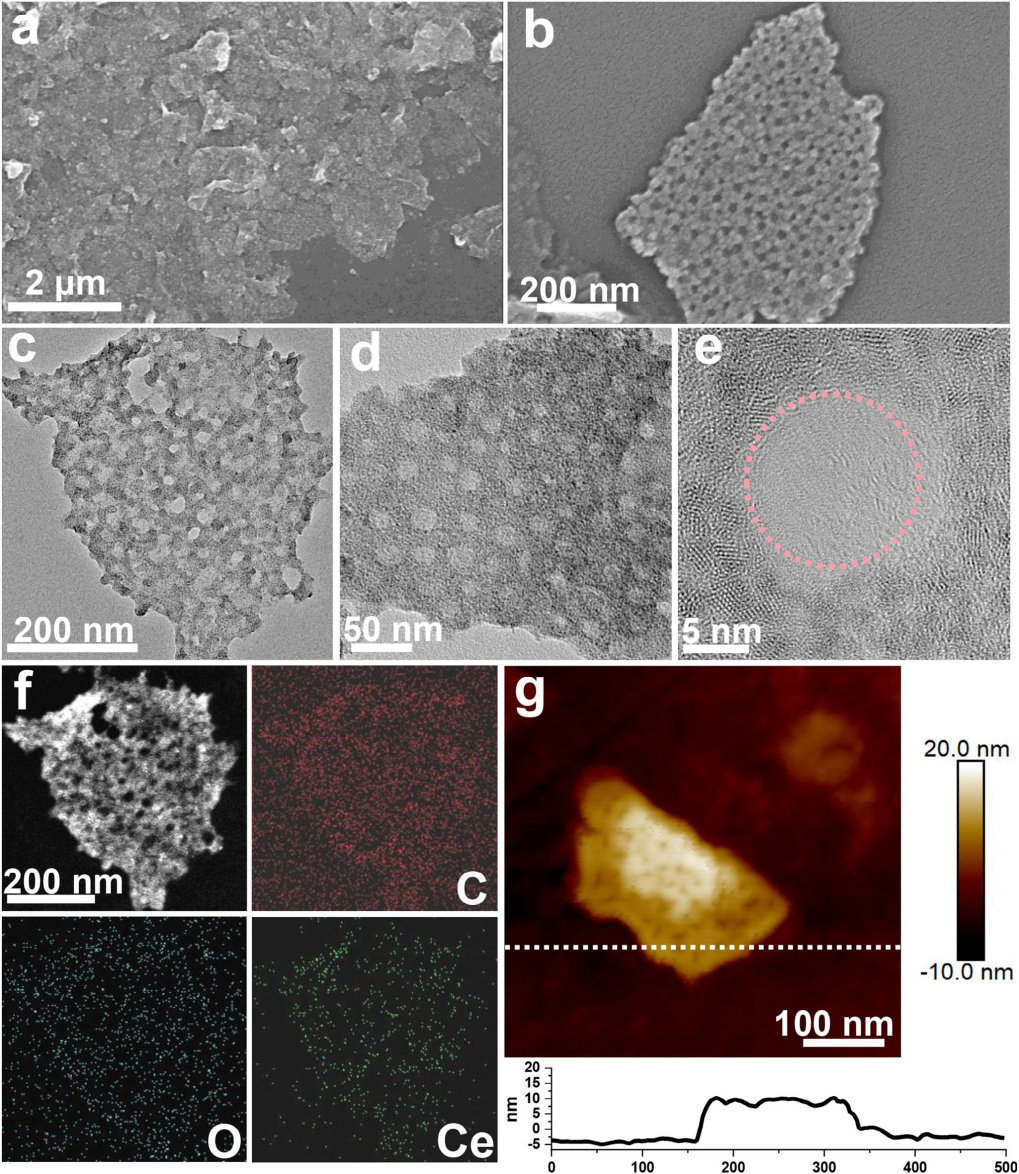
Morphological and structural characterizations of 2D ‐mUiO‐66(Ce). a,b) SEM images, c–e) TEM images, f) elemental mapping, and g) AFM image and its corresponding height information.

Scanning electron microscopy (SEM) and transmission electron microscopy (TEM) were used to examine the morphology and structure of 2D‐mUiO‐66(Ce). The resulting 2D‐mUiO‐66(Ce) displays a unique sheet‐like morphology, and the lateral size of these nanosheets can be extended to the micrometer scale (**Figure** **2**a,b). The formation of uniformly sized and permeable mesopores has an average size of ≈23 nm (Figure S2, Supporting Information), which is consistent with the TEM observation (Figure 2c). The TEM images (Figure 2d,e) further demonstrate that the monolayered mesopores are evenly distributed across the nanosheets. It is clearly observed that individual mesopores pass through the nanosheet thickness. The elemental mapping images reveal a uniform distribution of Ce, C, and O in the 2D‐mUiO‐66(Ce) (Figure 2f). The obtained nanosheets are well dispersed in the suspension. A portion of the sample suspension was transferred onto a solid substrate for atomic force microscopy (AFM) analysis. The AFM height profile indicates that the nanosheets have a thickness of ≈13 nm (Figure 2g).

The thickness of 2D MOF nanosheets can be finely controlled by adjusting the quantity of organic linkers, BDC, used during synthesis. Reducing the amount of organic linkers decreases the thickness of 2D mUiO‐66(Ce)‐1 to ≈3 nm (Figure S3b, Supporting Information). Conversely, increasing the amount of organic linkers results in a thickness of ≈150 nm for 2D mUiO‐66(Ce)‐2 (Figure S4b, Supporting Information). A series of SEM images reveal no significant morphological changes with varying BDC dosage, and uniform mesopores are consistently observed in all 2D mUiO‐66(Ce) samples (Figures S3,S4, Supporting Information). To clarify the role of the PFCA‐derived lipid bilayers, when PFCA is not used, cubic‐shaped mesoporous UiO‐66(Ce) (abbreviated as mUiO‐66(Ce)) particles with uniform mesopores on the surface are formed (Figure S5a,b, Supporting Information). The TEM image of mUiO‐66(Ce) (Figure S5c, Supporting Information) reveals numerous mesopores throughout the cubic particles. This study highlights the importance of lipid bilayers in the development of 2D nanosheets. To explain the role of PS‐*b*‐PEO, the control experiments were carried out by varying the reaction parameters. When PFCA was used alone without PS‐*b*‐PEO, no UiO‐66 product was obtained, indicating that PS‐*b*‐PEO plays a crucial role in initiating MOF nucleation. This effect is likely due to hydrogen bonding interactions between the PEO segments and the Ce precursor, which guide the initial coordination and promote anisotropic growth. These results highlight the synergistic effect among PFCA, PS‐*b*‐PEO, and the Ce‐based MOF precursors, which collectively direct the formation of the unique 2D mesoporous architecture. For comparison in later application tests, conventional UiO‐66(Ce) particles with smooth surfaces and without mesopores were prepared according to a general synthetic process (Figure S6, Supporting Information).

**Figure 3:**
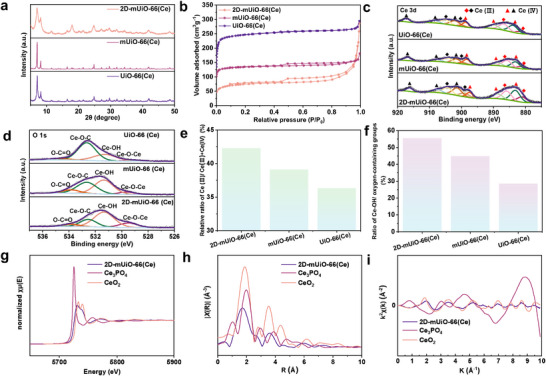
a) XRD patterns, b) N_2_ adsorption‐desorption isotherms, c) Ce 3d XPS patterns, d) O 1s XPS patterns, e) percentage of Ce (III)/ Ce (III)+Ce (IV), f) percentage of Ce‐OH/oxygen‐containing groups, g) X‐ray absorption near‐edge spectra (XANES), h) Fourier transforms of EXAFS spectra, i) k^2^‐weighted Ce K‐edge EXAFS of 2D‐mUiO‐66(Ce), Ce_3_PO_4_, and CeO_2_.

The peak locations in the wide‐angle X‐ray diffraction (XRD) patterns of 2D‐mUiO‐66(Ce) and mUiO‐66(Ce) closely match those of conventional UiO‐66(Ce) with a face‐centered cubic topology (**Figure** **3**a). In contrast, for 2D‐mUiO‐66(Ce), while the peak positions remain constant, each peak broadens and exhibits lower relative intensity, suggesting a decrease in crystallinity due to structural defects from the confined MOF growth within the 2D templates. The FT‐IR spectra (Figure S7a, Supporting Information) show two characteristic peaks at 1552 and 1388 cm^−1^, corresponding to the stretching vibrations of the ─COO^−^ groups in the 2D‐mUiO‐66(Ce) structure. Additionally, the peaks at 584 and 520 cm^−1^ are assigned to the Ce─(OC) and Ce─OH stretching vibrations, respectively. Other peaks observed at 1152 and 1207 cm^−1^ are attributed to the stretching vibrations of the fluorocarbon sequence (─CF_2_─)_n_ present in PFCA (Figure S8, Supporting Information). This may be because the carboxyl group in PFCA likely interacts with the secondary building units during crystallization, acting as a coordination modulator that regulates the nucleation and growth of the 2D‐mUiO‐66(Ce) framework. The Raman spectra (Figure S7b, Supporting Information) display peaks at 1138, 1420, and 1612 cm^−1^, which are attributed to the C─H bending vibrations of the terephthalic acid (H_2_BDC) ligand, while the peaks at 422 and 850 cm^−1^ is assigned to the Ce─O─Ce and Ce─OC vibrations in the metal clusters.^[^ ^32^ ^]^

In contrast to the type I isotherms observed for conventional UiO‐66(Ce), the N₂ adsorption‐desorption isotherms for 2D‐mUiO‐66(Ce) and mUiO‐66(Ce) both show a gradual uptake of N₂ gas with pronounced hysteresis loops in the high‐pressure range of 0.40 < *P*/*P*₀ < 1.0 (Figure 3b), indicating the presence of a substantial mesoporous structure.^[^ ^33^ ^]^ The estimated BET‐specific surface area and total pore volume of 2D‐mUiO‐66(Ce) are 278.9 m^2^ g^−1^ and 0.403 cm^3^ g^−1^, respectively. In comparison, the estimated BET‐specific surface areas for mUiO‐66 (Ce) and UiO‐66 (Ce) are 503.32 and 892.9 m^2^ g^−1^, respectively, with total pore volumes of 0.261 and 0.449 cm^3^ g^−1^. It is generally known that surface area decreases gradually with increasing pore size. Therefore, the surface areas of 2D‐mUiO‐66(Ce) and m‐UiO‐66(Ce), which contain micro‐mesopores, are smaller than those of UiO‐66(Ce) with predominantly micropores. The decrease in the surface area of 2D‐mUiO‐66(Ce) relative to mUiO‐66(Ce) is attributed to structural defects resulting from a reduction in the crystallinity of the MOF framework, as identified by XRD (Figure 3a).

X‐ray photoelectron spectroscopy (XPS) analysis for Ce 3*d* reveals the significant presence of Ce^4+^/Ce^3+^ clusters in 2D‐mUiO‐66(Ce), mUiO‐66(Ce), and UiO‐66(Ce) (Figure 3c).^[^ ^34^ ^]^ The peaks marked with black triangles at 916.2, 907.6, and 901.4 eV are associated with the core hole 3*d*_3/2_ signals of Ce(IV). The peaks indicated by the red triangles at 897.5, 888.7, and 883.2 eV correspond to the spin‐orbit split 3*d*_5/2_ signals of Ce(IV). Black diamond symbols at 904.4 and 899.1 eV denote core hole 3*d*_3/2_ signals of Ce(III), while red diamonds at 885.9 and 881.1 eV signify the spin‐orbit split 3*d*_5/2_ signals of Ce(III).^[^ ^35^ ^]^ A comparison of the bulk UiO‐66 (Ce) with the XPS spectrum of 2D‐mUiO‐66 (Ce) reveals a significant increase in the proportion of Ce(III) from 36.3% to 42.3% (Figure 3e). This observation suggests a transition from coordination‐unsaturated Ce(IV)‐OH sites to Ce(III) sites within the prominently exposed Ce_6_O_4_(OH)_4_ clusters of 2D‐mUiO‐66 (Ce).

The O 1*s* spectra for 2D‐mUiO‐66 (Ce), mUiO‐66 (Ce), and UiO‐66 (Ce) display four distinct peaks at 533.9, 532.5, 531.3, and 529.5 eV (Figure 3d). These peaks are attributed to oxygen in uncoordinated (O─C═O) and coordinated carboxylate groups (Ce‐O‐C), cerium hydroxyl active sites lacking full saturation (Ce‐OH), and bridging oxygen bonds within the Ce_6_ clusters (Ce‐O‐Ce).^[^ ^36^ ^]^ Significantly, 2D‐mUiO‐66(Ce) has a higher proportion of Ce‐OH sites (55.4%) than bulk UiO‐66 (Ce) (28.5%) and mUiO‐66 (Ce) (44.9%), indicating that more Ce_6_O_4_(OH)_4_ clusters were exposed on its surface because of its 2D structure (Figure 3f). This observation is consistent with the increased presence of Ce(III) detected in the Ce 3*d* XPS analysis.

The X‐ray absorption near‐edge structure (XANES) spectrum of 2D‐mUiO‐66(Ce) (Figure 3g) reveals a noticeable shift of the absorption edge toward lower energy compared to CeO_2_, a standard Ce^4+^ reference. This shift suggests the partial reduction of Ce^4+^ to Ce^3+^. The spectral position is closer to that of Ce_3_PO_4_, which predominantly contains Ce^3+^, indicating that Ce in the MOF framework exists in a mixed‐valence state. Further insights are obtained from the Fourier‐transformed EXAFS spectra (Figure 3h).^[^ ^37^ ^]^ The first shell peak in 2D‐mUiO‐66(Ce) appears at a slightly shorter radial distance and exhibits a significantly lower intensity than those of Ce_3_PO_4_ and CeO_2_. This attenuation and broadening of the peak imply a more disordered and under‐coordinated local environment, consistent with the presence of Ce^3+^, which typically forms longer and more variable Ce─O bonds. In the k‐space EXAFS spectra (Figure 3i), 2D‐mUiO‐66(Ce) displays a markedly dampened oscillation amplitude across the entire k‐range, relative to the reference compounds. This damping is indicative of reduced scattering strength and structural disorder, further supporting a lower oxidation state and coordination number. Altogether, the XANES edge shift, the weakened and broadened FT‐EXAFS peaks, and the suppressed χ(k) oscillations provide consistent evidence for the partial reduction of Ce^4+^ to Ce^3+^ in 2D‐mUiO‐66(Ce). This mixed‐valence state likely contributes to framework flexibility and may enhance the material's redox and adsorption capabilities.

**Figure 4:**
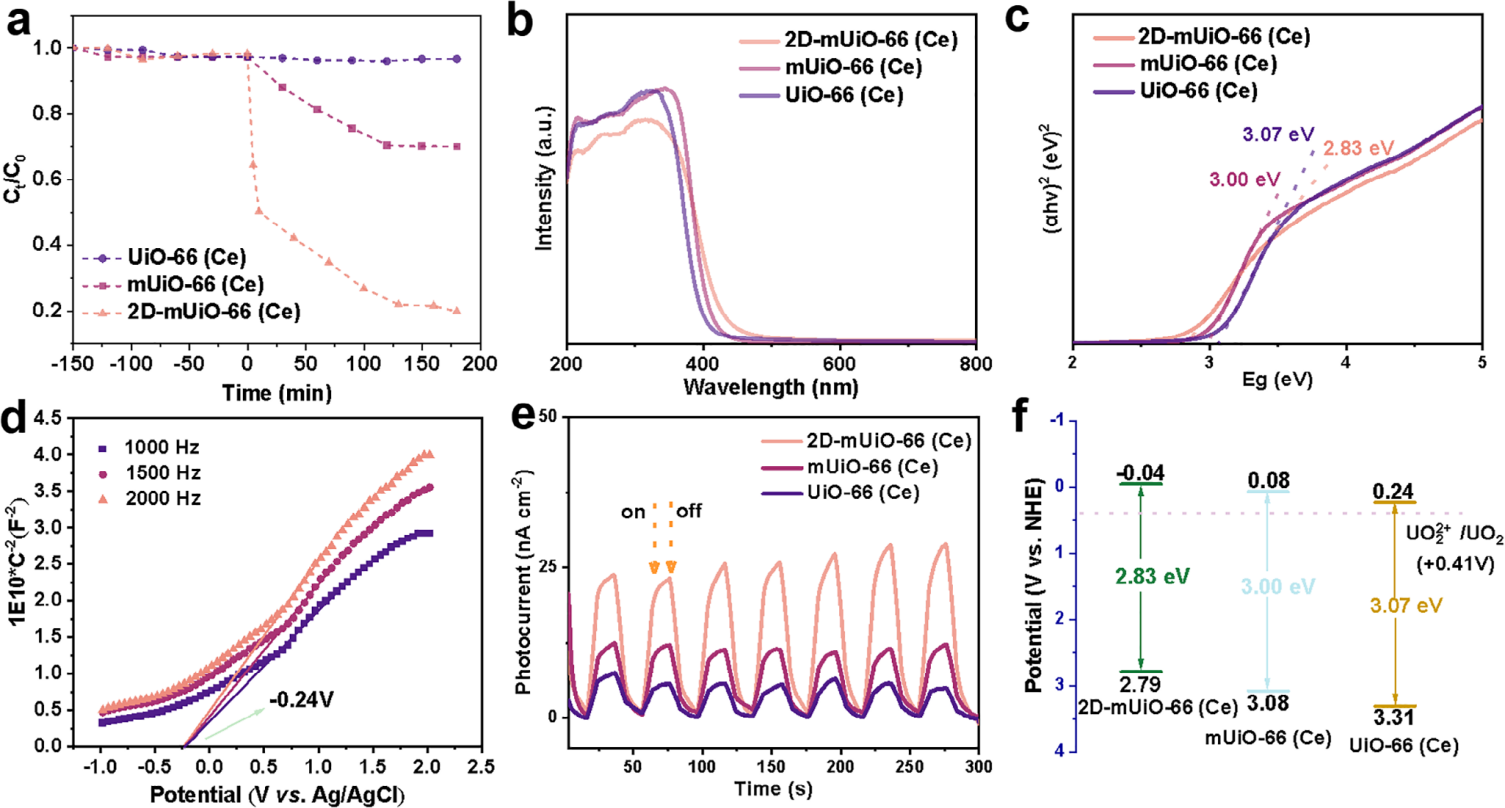
a) U(VI) (50 ppm) removal ratio in the dark and upon irradiation, b) UV–vis adsorption spectra, c) Tauc plot for band gap calculation, d) Mott–Schottky plots, e) transient current density, and f) band structure alignments.

The photocatalytic performance of 2D‐mUiO‐66 (Ce) in reducing U(VI) was evaluated in an aqueous solution with 50 ppm U(VI) and methanol as sacrificial agents under air, employing a solid‐to‐liquid ratio of 0.1 g L^−1^ to demonstrate proof of concept. The 2D‐mUiO‐66(Ce) sample with a thickness of 13 nm exhibited better performance than the other two samples, 2D‐mUiO‐66(Ce)‐1 and 2D‐mUiO‐66(Ce)‐2 (Figure S9, Supporting Information). For comparison, both solid UiO‐66(Ce) (with predominant micropores) and mesoporous UiO‐66(Ce) (with hierarchical micro‐mesopores), both of which are in particle form, were tested under similar conditions. As shown in **Figure** **4**a, after 150 min in the dark, 2D‐mUiO‐66 (Ce), mUiO‐66 (Ce), and UiO‐66 (Ce) reach adsorption equilibrium with similar adsorption capacities and an approximate removal efficiency of 2%. Upon exposure to light, the U(VI) removal efficiency of 2D‐mUiO‐66(Ce) increases significantly to 80.3%. This efficiency exceeds that of mUiO‐66 (30.0%), highlighting superior performance for photoinduced U(VI) removal. To assess the reaction kinetics, pseudo‐first‐order kinetic models are employed to determine the photoreaction rate constants (*k*) for the different photocatalysts. The rate constants (Figure S10, Supporting Information) are ranked as follows: 2D‐mUiO‐66(Ce) > mUiO‐66(Ce) > UiO‐66(Ce). Consequently, 2D‐mUiO‐66 (Ce) demonstrates enhanced photocatalytic activity compared to the other photocatalysts, confirming the advantages of its 2D mesoporous architecture. The 2D‐mUiO‐66(Ce) photocatalyst shows effective photoreduction of U(VI) at an initial concentration of 50 ppm, as shown in Figure 4a. As the initial concentration of U(VI) increases, the reduction efficiency decreases, although the performance for photoinduced U(VI) removal remains high (Figure S11, Supporting Information). This reduction in efficiency is due to the increased coverage of U(VI) on the 2D‐mUiO‐66(Ce) photocatalyst, which hinders the absorption of light.

UV–vis data demonstrate that all the samples exhibit similar absorption profiles between 200 and 800 nm (Figure 4b). Furthermore, the optical bandgap (*E*_g_) of 2D‐mUiO‐66 (Ce), calculated using the Tauc plot equation, is 2.83 eV, which is narrower than those of mUiO‐66(Ce) (3.00 eV) and UiO‐66(Ce) (3.07 eV) (Figure 4c). The narrower bandgap of 2D‐mUiO‐66(Ce) implies a lower energy requirement for photocatalytic reactions.^[^ ^38^ ^]^ Electrochemical Mott–Schottky measurement identifies the flat‐band potential, further elucidating the energy band structure. The positive slopes indicate that 2D‐mUiO‐66(Ce), mUiO‐66(Ce), and UiO‐66(Ce) are typical *n*‐type semiconductors.^[^ ^39^ ^]^ Given the proximity of the flat band potential to the conduction band, the *E*_CB_ values are ‐0.24 V (Figure 4d), −0.124, and 0.073 V versus Ag/AgCl (i.e., −0.04, 0.08, and 0.24 V versus NHE) for 2D‐mUiO‐66 (Ce), mUiO‐66 (Ce), and UiO‐66 (Ce), respectively. The *E*_VB_ values calculated using the *E*_g_ = *E*_VB_−*E*_CB_ equation are 2.79, 3.08, and 3.31 V versus NHE. Figure 4e shows that 2D‐mUiO‐66 (Ce) has a significantly higher transient current density than mUiO‐66(Ce) and UiO‐66(Ce), indicating rapid interfacial charge transfer.^[^ ^40^ ^]^ The PL intensity (Figure S12, Supporting Information) of 2D‐mUiO‐66(Ce) is significantly lower than that of mUiO‐66(Ce) and UiO‐66(Ce). These results indicate that 2D‐mUiO‐66(Ce) more effectively enhances the spatial separation of photogenerated charge carriers and prevents electron–hole recombination compared to the other two samples. The narrower semicircular diameter in the Nyquist plot of 2D‐mUiO‐66 (Ce) (Figure S13, Supporting Information) compared to the other samples suggests a reduced interfacial charge transport resistance and enhanced charge transfer and photocatalytic activity.^[^ ^41^ ^]^ The band structure alignments, shown in Figure 4f, highlight that the E_CB_ of 2D‐mUiO‐66(Ce) is more negative than the U(VI)/U(IV) redox potential (0.41 V versus NHE), demonstrating its thermodynamic advantage in reducing U(VI) to U(IV).^[^ ^42^ ^]^ To evaluate the structural stability and durability of the catalyst, SEM observation was carried out after the photocatalytic reaction. As shown in Figure S14 (Supporting Information), the catalyst retains its original morphology without significant structural degradation or collapse, indicating good structural robustness during the reaction process. These results confirm the catalyst's durability under photocatalytic conditions. The outstanding photocatalytic efficiency of 2D‐mUiO‐66(Ce) for the reduction of U(VI) arises from multiple critical aspects. First, the through‐pores of its 2D structure, which are absent in both UiO‐66(Ce) and mUiO‐66(Ce), shorten the diffusion path, thereby improving mass transport and the availability of active sites. Second, the elevated levels and uniform distribution of the Ce(III)‐enriched Ce_6_O_4_(OH)_4_ clusters within 2D‐mUiO‐66(Ce) significantly contribute to its enhanced photocatalytic performance.

**Figure 5:**
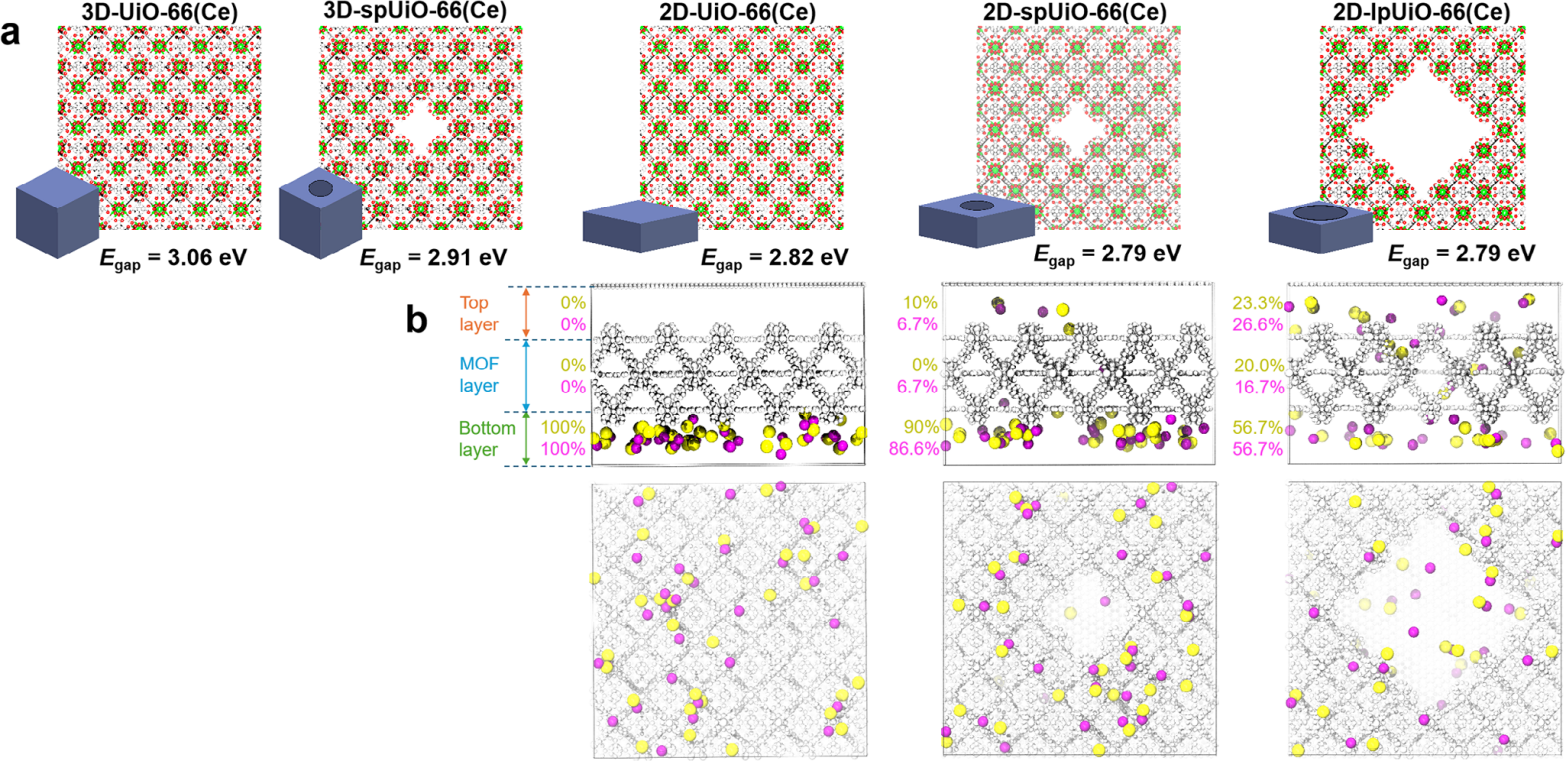
a) Top view of the models investigated with DFT: 3D‐UiO‐66(Ce), 3D‐spUiO‐66(Ce), 2D‐UiO‐66(Ce), 2D‐spUiO‐66(Ce), and 2D‐lpUiO‐66(Ce), where “sp” denotes small‐pore and “lp” denotes large‐pore. Green, red, and white spheres represent Ce, O, and C atoms, respectively. The calculated band gap (in eV) for each model is also shown. b) Snapshots from MD simulations at 30 ns, showing both side and top views. Pink and yellow spheres represent Na^+^ and Cl^−^ ions, respectively, while MOF atoms are shown in white for clarity. The percentage values indicate the proportion of ions in the bottom, MOF, and top layers.

Large‐scale density functional theory (DFT) calculations were performed to confirm the experimental findings on the reduction of the band gap from UiO‐66(Ce) to mUiO‐66(Ce) and 2D‐mUiO‐66(Ce). To this end, we compute the band gaps of five structural models, as shown in **Figure** **5a**: 3D‐UiO‐66(Ce), 3D‐spUiO‐66(Ce), 2D‐UiO‐66(Ce), 2D‐spUiO‐66(Ce), and 2D‐lpUiO‐66(Ce), where “sp” and “lp” denote small‐pore and large‐pore structures, respectively. The theoretical results corroborate the experimental observations, revealing a systematic decrease in band gap with increasing structural modifications. For 3D‐UiO‐66(Ce), the calculated band gap is 3.06 eV, being in close agreement with the experimental optical band gap of 3.07 eV (Figure 4f) and a previous theoretical prediction of 2.66 eV.^[^ ^43^ ^]^ The introduction of a small pore in the 3D structure reduces the band gap to 2.91 eV, a decrease of 0.15 eV, consistent with the observed band gap reduction in mUiO‐66(Ce) (3.00 eV). The most significant decrease occurs upon transitioning from a 3D to a 2D framework, as seen in the 2D‐UiO‐66(Ce) model, where the band gap drops further to 2.82 eV. However, introducing additional porosity in the 2D structures (sp‐2D and lp‐2D) results in only a marginal further reduction, with both models exhibiting a band gap of 2.79 eV. This value is in excellent agreement with the experimentally measured optical band gap of 2.83 eV for 2D‐mUiO‐66(Ce). Overall, the theoretical results indicate that the primary factor driving the band gap reduction should be the transition from a 3D to a 2D structure, rather than the formation of pores.

While the presence of pores may have a small effect on the band gap, it plays a crucial role in mass transport, which is essential for enhancing the kinetics of chemical reactions. To investigate this, we performed molecular dynamics (MD) simulations of ion transport through three 2D‐UiO: 2D‐UiO‐66(Ce), 2D‐spUiO‐66(Ce), and 2D‐lpUiO‐66(Ce); using Na^+^ and Cl^−^ as model ions (Figure 5b). The simulations were done similarly to our previous work.^[^ ^44^ ^]^ Initially, 30 Na^+^ and 30 Cl^−^ ions were placed in the bottom layer of the simulation box. The systems were equilibrated at 300 K and the simulations were run for 30 ns. At the end of the simulation, no ions had diffused into the MOF or top layers in 2D‐UiO‐66(Ce), indicating extremely slow ion transport. This limited diffusion is likely due to the absence of pores as well as the modest rotational barrier of the linker,^[^ ^45^, ^46^ ^]^ which controls the gate‐opening mechanism. Introducing small pores (2D‐spUiO‐66(Ce)) modestly improves the diffusion rate, with ≈10–12% of ions diffusing into the MOF and top layers by 30 ns. Doubling the pore size (2D‐lpUiO‐66(Ce)) led to a significant enhancement of charge transport, with ≈50% of ions migrating from the bottom to the MOF and top layer. The results from our MD simulations clearly demonstrate that porosity substantially enhances mass transport, which is a critical factor in optimizing reaction kinetics in porous materials.

## 3 Conclusion

A pioneering bottom‐up method has been successfully introduced for the synthesis of ultrathin 2D mesoporous MOF nanosheets, 2D‐mUiO‐66(Ce). This novel approach utilizes the interface‐directed co‐assembly of 2D self‐assembled lipid bilayers from amphiphilic perfluorooctanoic acid and spherical micelles from PS‐*b*‐PEO block copolymers. This results in the formation of 2D hierarchical structures resembling the configuration of sandwiches, with spherical micelles systematically arranged in a monolayer on the 2D bilayers. Based on these assemblies, the MOFs are grown in confined spaces, forming ultrathin mesoporous MOF nanosheets. Notably, the resulting 2D‐mUiO‐66(Ce) exhibits superior U(VI) photoreduction efficiency compared to solid UiO‐66(Ce) (with predominant micropores) and mesoporous UiO‐66(Ce) (with hierarchical micro‐mesopores), both of which are in particle form. This is attributed to enhanced mass transport, uniform distribution of catalytically active sites, and optimal band structures. This breakthrough represents a substantial contribution to the development of more efficient and effective MOF materials for environmental remediation and energy applications, highlighting the significance of novel material synthesis techniques in advancing the capabilities of 2D mesoporous materials.

## 4 Experimental Section

### Preparation of 2D‐m‐UiO‐66(Ce)

2 mL of THF was used to fully dissolve 20 mg of PS_9500_‐*b*‐PEO_5000_ diblock copolymers in a standard optimized synthesis. Subsequently, 0.8 mL of water was gradually added with continuous magnetic stirring. The 400 µL PFCA ethanol solution (12 mg mL^−1^) was added. After a period of stirring, 40 µL of AA and 40 mg of NaClO_4_ were introduced. The mixture was then stirred until homogeneous. Upon adding 218 mg of (NH_4_)_2_Ce(NO_3_)_6_ and 66 mg of BDC, the mixture was stirred at 60 °C for 15 min. The resulting solid was collected by centrifugation and washed with water, THF, EtOH, and DMF. Finally, the product was dried under vacuum at 60 °C overnight to complete the process. 2D mUiO‐66 (Ce)‐1 and 2D mUiO‐66 (Ce)‐2 were obtained by changing the amount of BDC to 33 and 198 mg, respectively.

### Preparation of m‐UiO‐66(Ce)

2 mL of THF was used to fully dissolve 20 mg of PS_9500_‐*b*‐PEO_5000_ diblock copolymers in a standard optimized synthesis. Subsequently, 0.8 mL of water was gradually added with continuous magnetic stirring. After a period of stirring, 40 µL of AA and 40 mg of NaClO_4_ were introduced. The mixture was then stirred until homogeneous. Upon adding 218 mg of (NH_4_)_2_Ce(NO_3_)_6_ and 66 mg of BDC, the mixture was stirred at 60 °C for 15 min. The resulting solid was collected by centrifugation and washed with water, THF, and DMF. Finally, the product was dried under vacuum at 60 °C overnight to complete the process.

### Preparation of UiO‐66(Ce)

UiO‐66(Ce) was prepared by dissolving 35.4 mg of BDC in 1.2 mL of DMF, followed by the addition of 400 µL of 0.5 m (NH_4_)_2_Ce(NO_3_)_6_ solution, conducted at 100 °C for 15 min.

### Photocatalytic Reduction of U(VI)

Experiments to assess the photocatalytic reduction of U(VI) took place in a 200 mL photoreactor, under light in an air‐filled environment. A 300 W xenon lamp (PerfectLight, PLS‐SXE300D) was used for illumination. The experimental setup involved combining a catalyst sample (5 mg), a U(VI) solution (45 mL, 50 ppm), and 5 mL of CH_3_OH in a glass reaction vessel. Throughout the reaction, samples were periodically collected, and filtered through a 0.45 µm membrane filter, and the U(VI) concentration in the filtrate was measured by UV–vis spectrophotometry using the Arsenazo III method at 652 nm. For the U(VI) adsorption studies, the same procedure was followed except the xenon lamp did not illuminate the mixture. A specific equation was used to calculate the photocatalytic reaction's rate constant, the amount of U(VI) removed, and the removal efficiency:

(1) $$\mathrm{Removal}\;\mathrm{ratio}=\left(C_{0}\;-\;C_{t}\right)/C_{0}\;\times \;100\%$$

(2) $$\mathrm{Extraction}\;\mathrm{mass}=(C_{0}\;-\;C_{t})\;\times \;V/m$$

(3) $$\mathrm{kt}=\ln\;(C_{0}/C_{t})$$

Where *C*_t_ is the time concentrations of U(VI) (mg L^−1^), *C*_0_ is the initial concentrations of U(VI) (mg L^−1^), V is the volume of the solution (L), t is the reaction time (min), and m is the sample mass (g).

### Characterization

The morphology of the sample was examined with a Hitachi S4800 scanning electron microscope at 10 kV acceleration voltage. TEM and EDX analyses were performed on a JEM‐2100F at 300 kV. X‐ray diffraction (XRD) measurements were conducted on a Rigaku Smart lab X‐ray diffractometer, using Cu‐Kα radiation at 40 kV and 30 mA, with a scanning rate of 5° min^−1^. The BELSORP MAX instrument measured the sample's specific surface area and pore size through N_2_ adsorption/desorption isotherms at 77.5 K, using the multi‐point BET method for adsorption data between relative pressures of 0.03 and 0.15. XPS (JPS‐9010) was evaluated with Al K radiation. EXAFS measurements were conducted in beamline TPS‐44A Quick‐scanning X‐ray Absorption Spectroscopy, NSRRC, Taiwan. FT–IR spectra were recorded using a JASCO FT/IR 4X. The inVia Reflex from Renishaw was used to study the structure of 2D‐mUiO‐66(Ce) at room temperature using Raman spectroscopy. Steady‐state photoluminescence (PL) was measured on FLS1000‐Edinburgh Instruments.

## Conflict of Interest

The authors declare no conflict of interest.

## Supporting information



Supporting Information

## Data Availability

The data that support the findings of this study are available from the corresponding author upon reasonable request.
