# Supplementary material for: Ultrathin Mesoporous Metal‐Organic Framework Nanosheets
Source: Adv Mater. 2025 Jun 16;37(36):2508105. doi: 10.1002/adma.202508105 (PMC12422086; doi:10.1002/adma.202508105)
Supplement: Supplementary file 1 — Supporting Information [file ADMA-37-2508105-s001.docx]

**Supporting Information**

**Computational Details**

**DFT Calculations**. To obtain the band gaps of the materials, we performed density functional theory (DFT) calculations using the OpenMX and Vienna Ab initio Simulation Package (VASP) codes.^[1–6]^ The crystal structure of UiO-66(Ce) was first obtained at the PBE-D3(BJ) level of theory,^[7,8]^ employing the projector-augmented wave (PAW) method,^[9,10]^ with a kinetic energy cutoff of 450 eV. The optimized lattice constant of UiO-66(Ce) was found to be 21.590 Å, in close agreement with the experimental value of 21.472 Å.^[11]^ At this theoretical level, the computed band gap was 1.807 eV. Alternatively, using a lattice optimized with the UFF4MOF force field (lattice constant of 21.571 Å), the calculated band gap increases only slightly to 1.811 eV (**Table S1**). These results indicate that small variations in the lattice constant have a negligible impact on the band gap. Based on this observation, we will proceed with geometries optimized with the UFF4MOF force field.^[12,13]^ This allows us to study larger porous systems containing more than 5000 atoms.

**Table S1.** Band gap (in eV) obtained with different lattice constants (in Å)

| Code | Optimized structure | Lattice constant (Å) | Band gap (eV) |
| --- | --- | --- | --- |
| VASP | DFT | 21.590 | 1.807 |
| VASP | UFF4MOF | 21.571 | 1.811 |
| OpenMX | UFF4MOF | 21.571 | 1.917 |

For large systems, we used a hybrid approach that combines an *O*(*N*) method and the conventional diagonalization method implemented in the OpenMX code. Specifically, the DC-LNO method^[14]^ was used to obtain a self-consistent charge density, after which the conventional diagonalization method was employed to obtain full wave functions under the self-consistent charge density. The 2019 database of optimized fully relativistic pseudopotentials (VPS) and pseudoatomic orbitals (PAO) was used. To reduce the computational cost, a minimal PAO basis set was adopted, consisting of H-s1, C-s1p1, O-s1p1, and Ce-s2p1d1f1. Despite this reduced basis, the computed band gap for 3D-UiO-66(Ce) remained in good agreement with that obtained using VASP.

It is well known that PBE underestimates band gaps, whereas the HSE functional has been shown to provide more accurate estimation for the MOF band gap. Thus, we applied a correction scheme proposed by Jin et al.^[15]^ that approximate HSE band gaps from PBE band gaps. For 3D systems, the corrected band gap was estimated using the relation
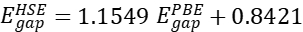
, whereas for 2D systems,
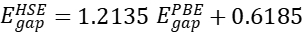
.

**Molecular Dynamics (MD) simulations**

MD simulations were performed with the LAMMPS package^[16]^ to explore the diffusion of Na^+^ and Cl^−^ in three 2D models: 2D-UiO-66(Ce), 2D-spUiO-66(Ce), and 2D-lpUiO-66(Ce) (**Figure 5**(b)). The first model was created by cleaving a (001) slab with a thickness of ~26 Å from the optimized 3D-UiO-66(Ce) structure. All dangling bonds were capped with hydrogen. To create a layered system, vacuum regions (~12 Å each) were introduced above and below the slab, forming a simulation box comprising three sections: bottom (*z* = 0–12 Å), MOF (*z* = 12–38 Å), and top (*z* = 38–50 Å). The total dimensions of the simulation box were 76.3 × 76.3 × 50.4 Å^3^. To investigate the impact of porosity, the 2D-spUiO-66(Ce) model was created by introducing a small pore at the center of the box, while the 2D-lpUiO-66(Ce) model featured a doubled pore size.

Each system was solvated using a pre-equilibrated TIP4P water reservoir. A total of 30 Cl^−^ and 30 Na^+^ ions were then placed in the bottom region. Any overlapping water molecules within 1.5 Å of the MOF and ions were removed. To ensure that ions diffused through the MOF rather than bypassing it, an immobile graphene barrier was placed at the top boundary of the simulation box. The UFF4MOF force field^[12,13,17,18]^ was used for the MOF framework, whereas the OPLSAA force field parameters were employed for Cl^−^ and Na^+^ ions.^[19–21]^ Following the initial model setup, each system underwent energy minimization, followed by a 30 ps equilibration phase in the microcanonical ensemble (NVE) using a Berendsen thermostat set at 300 K.^[22]^ Nonbonded interactions were treated using the lj/cut/tip4p/long pair potential with a 12 Å cutoff.^[23]^ Water molecules were treated using the SHAKE algorithm to constrain OH bond lengths and HOH angles.^[24]^ The production run was carried out for 30 ns in the canonical ensemble (NVT) with a Nosé-Hoover thermostat maintaining a temperature of 300 K.^[25,26]^ All simulations employed a 1 fs timestep.

**Figure S1.** The Tyndall effect using a laser pointer to prove the micellization of the polymer. Optical micrographs of (a) PS-*b*-PEO dissolved in THF, (b) PS-*b*-PEO dissolved in a mixture of THF and water, and (c) the solution after adding PFCA ethanol solution.

**Figure S2.** (a) SEM image, and (b) AFM images and corresponding height information of 2D-mUiO-66 (Ce).

**Figure S3.** (a) SEM image and (b) AFM images and corresponding height information of 2D-mUiO-66 (Ce)-1.

**Figure S4.** (a) SEM image and (b) AFM images and corresponding height information of 2D-mUiO-66 (Ce)-2

**Figure S5.** (a, b) SEM images and (c) TEM image (c) of cubic mesoporous UiO-66 (Ce).

**Figure S6.** (a, b) SEM images and (c) TEM image of UiO-66 (Ce)


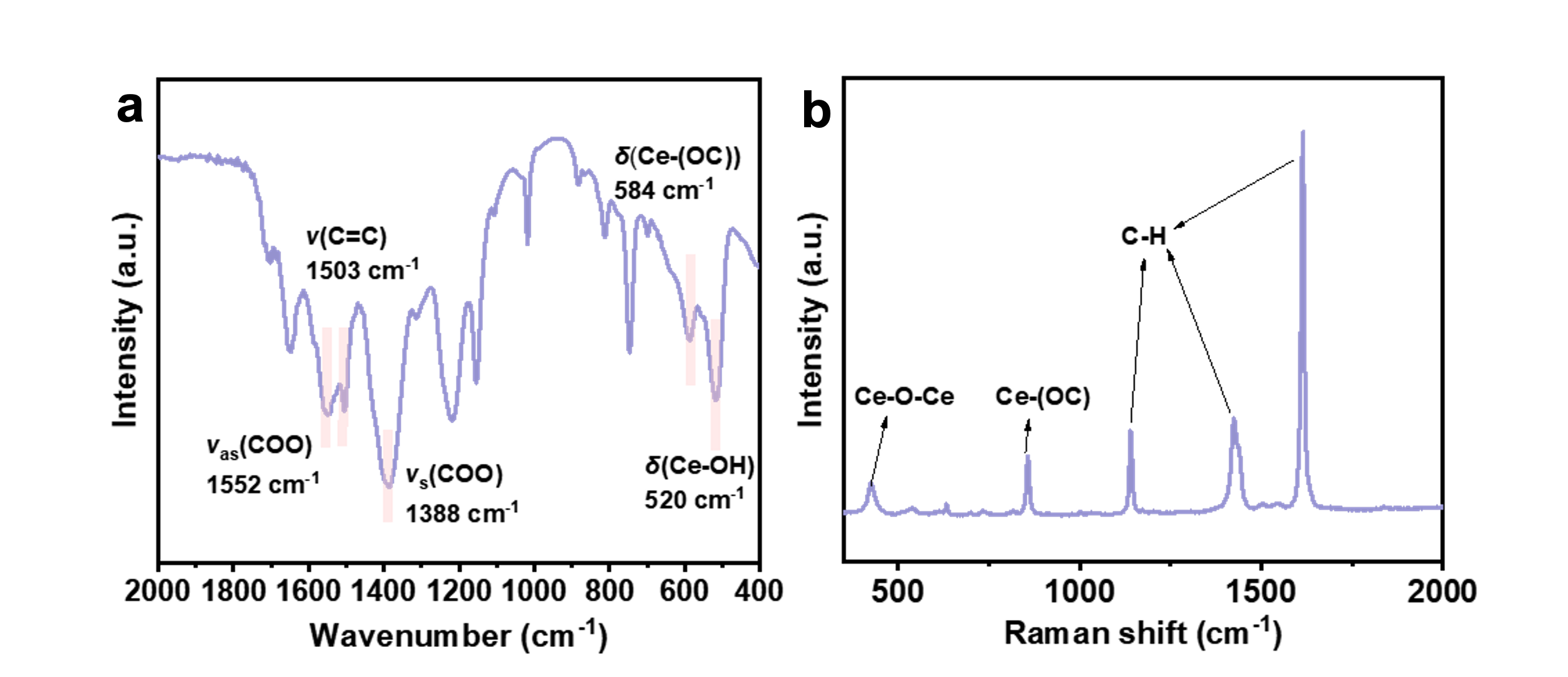


**Figure S7** (a) FT-IR spectra and (b) Raman spectra of 2D-mUiO-66(Ce).


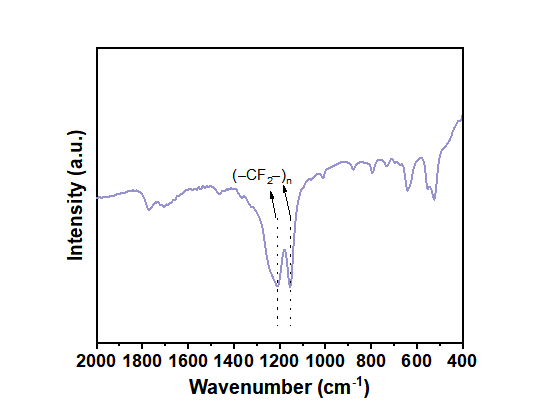


**Figure S8.** FT-IR spectra of PFCA

**Figure S9.** U(VI) (50 ppm) removal ratio in the dark and upon irradiation of 2D-mUiO-66(Ce)-1 and 2D-mUiO-66(Ce)-2


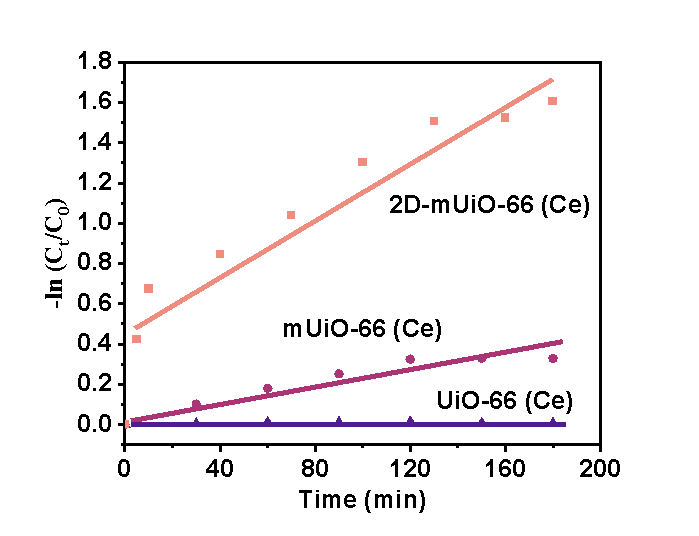


**Figure S10.** Reaction kinetics plots of U(VI) photoreduction

**Figure S11.** U(VI) removal ratio of 2D-mUiO-66 (Ce) in different concentrations of U(VI).

**Figure S12.** Steady-state PL spectra of samples


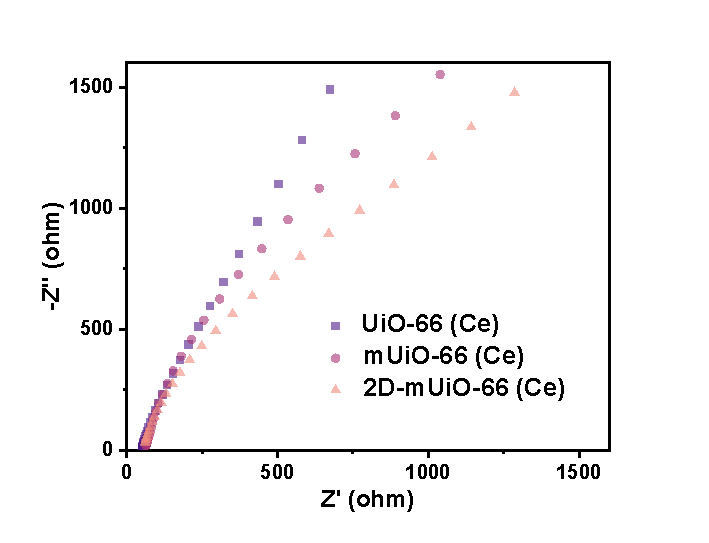


**Figure S13.** Nyquist plots of all samples

**Figure S14.** SEM images of 2D-mUiO-66(Ce) after reaction

**References**

[1] G. Kresse, J. Furthmüller, *Comput Mater Sci* **1996**, *6*, 15.

[2] G. Kresse, J. Hafner, *Phys Rev B* **1993**, *47*, 558.

[3] T. Ozaki, *Phys Rev B Condens Matter Mater Phys* **2003**, *67*, DOI 10.1103/PhysRevB.67.155108.

[4] T. Ozaki, H. Kino, *Phys Rev B Condens Matter Mater Phys* **2005**, *72*, DOI 10.1103/PhysRevB.72.045121.

[5] T. Ozaki, H. Kino, *Phys Rev B Condens Matter Mater Phys* **2004**, *69*, DOI 10.1103/PhysRevB.69.195113.

[6] K. Lejaeghere, G. Bihlmayer, T. Björkman, P. Blaha, S. Blügel, V. Blum, D. Caliste, I. E. Castelli, S. J. Clark, A. Dal Corso, S. De Gironcoli, T. Deutsch, J. K. Dewhurst, I. Di Marco, C. Draxl, M. Dułak, O. Eriksson, J. A. Flores-Livas, K. F. Garrity, L. Genovese, P. Giannozzi, M. Giantomassi, S. Goedecker, X. Gonze, O. Grånäs, E. K. U. Gross, A. Gulans, F. Gygi, D. R. Hamann, P. J. Hasnip, N. A. W. Holzwarth, D. Iuşan, D. B. Jochym, F. Jollet, D. Jones, G. Kresse, K. Koepernik, E. Küçükbenli, Y. O. Kvashnin, I. L. M. Locht, S. Lubeck, M. Marsman, N. Marzari, U. Nitzsche, L. Nordström, T. Ozaki, L. Paulatto, C. J. Pickard, W. Poelmans, M. I. J. Probert, K. Refson, M. Richter, G. M. Rignanese, S. Saha, M. Scheffler, M. Schlipf, K. Schwarz, S. Sharma, F. Tavazza, P. Thunström, A. Tkatchenko, M. Torrent, D. Vanderbilt, M. J. Van Setten, V. Van Speybroeck, J. M. Wills, J. R. Yates, G. X. Zhang, S. Cottenier, *Science (1979)* **2016**, *351*, DOI 10.1126/science.aad3000.

[7] J. P. Perdew, K. Burke, M. Ernzerhof, *Phys Rev Lett* **1996**, *77*, 3865.

[8] S. Grimme, S. Ehrlich, L. Goerigk, *J Comput Chem* **2011**, *32*, 1456.

[9] P. E. Blöchl, *Phys Rev B* **1994**, *50*, 17953.

[10] G. Kresse, D. Joubert, *Phys Rev B* **1999**, *59*, 1758.

[11] M. Lammert, M. T. Wharmby, S. Smolders, B. Bueken, A. Lieb, K. A. Lomachenko, D. De Vos, N. Stock, *Chemical Communications* **2015**, *51*, 12578.

[12] P. G. Boyd, S. M. Moosavi, M. Witman, B. Smit, *J Phys Chem Lett* **2017**, *8*, 357.

[13] M. A. Addicoat, N. Vankova, I. F. Akter, T. Heine, *J Chem Theory Comput* **2014**, *10*, 880.

[14] T. Ozaki, M. Fukuda, G. Jiang, *Phys Rev B* **2018**, *98*, DOI 10.1103/PhysRevB.98.245137.

[15] T. Wang, X. Tan, Y. Wei, H. Jin, *Mater Today Commun* **2021**, *29*, DOI 10.1016/j.mtcomm.2021.102932.

[16] A. P. Thompson, H. M. Aktulga, R. Berger, D. S. Bolintineanu, W. M. Brown, P. S. Crozier, P. J. in ’t Veld, A. Kohlmeyer, S. G. Moore, T. D. Nguyen, R. Shan, M. J. Stevens, J. Tranchida, C. Trott, S. J. Plimpton, *Comput Phys Commun* **2022**, *271*, 108171.

[17] C. Campañá, B. Mussard, T. K. Woo, *J Chem Theory Comput* **2009**, *5*, 2866.

[18] G. Zhao, Y. G. Chung, *J Chem Theory Comput* **2024**, *20*, 5368.

[19] L. S. Dodda, J. Z. Vilseck, J. Tirado-Rives, W. L. Jorgensen, *J Phys Chem B* **2017**, *121*, 3864.

[20] L. S. Dodda, I. Cabeza de Vaca, J. Tirado-Rives, W. L. Jorgensen, *Nucleic Acids Res* **2017**, *45*, W331.

[21] W. L. Jorgensen, J. Tirado-Rives, *Proceedings of the National Academy of Sciences* **2005**, *102*, 6665.

[22] H. J. C. Berendsen, J. P. M. Postma, W. F. van Gunsteren, A. DiNola, J. R. Haak, *J Chem Phys* **1984**, *81*, 3684.

[23] T. Darden, D. York, L. Pedersen, *J Chem Phys* **1993**, *98*, 10089.

[24] J.-P. Ryckaert, G. Ciccotti, H. J. C. Berendsen, *J Comput Phys* **1977**, *23*, 327.

[25] W. G. Hoover, *Phys Rev A  (Coll Park)* **1985**, *31*, 1695.

[26] S. Nosé, *J Chem Phys* **1984**, *81*, 511.
